# Carvedilol-loaded self-nanoemulsifying drug delivery system target diabetic nephropathy: preclinical evidence of antioxidant and antifibrotic effects

**DOI:** 10.1007/s00210-025-04356-9

**Published:** 2025-06-18

**Authors:** Hanan Elimam, Zeinab Hassan, Nora A. A. Alhamshry, Khalid M. El-Say, Ahmed M. A. Akabawy

**Affiliations:** 1 Department of Biochemistry, Faculty of Pharmacy, University of Sadat City, Sadat City, 32897 Egypt; 2 Department of Biochemistry and Molecular Biology, Faculty of Pharmacy, Helwan University, Ain Helwan 11795, Helwan, Cairo, Egypt; 3 Department of Pharmaceutics, Faculty of Pharmacy, King Abdulaziz University, 21589 Jeddah, Saudi Arabia

**Keywords:** Self-nanoemulsifying drug delivery system, Diabetic nephropathy, Carvedilol, Oxidative stress, Renal fibrosis

## Abstract

Diabetic nephropathy (DN) is a significant complication of diabetes, leading to chronic kidney failure and end-stage renal disease. While traditional treatments exist, innovative strategies are being investigated to enhance DN management. This study examines a novel nanosized formulation of carvedilol (CVL), a drug recognized for its β-blocker and α-1-adrenoreceptor antagonist properties. We developed a self-nanoemulsifying drug delivery system (SNEDS) to assess its potential in improving DN outcomes. DN was induced in rats using streptozotocin (STZ), and the animals received oral administration of either CVL or CVL-SNEDS for 6 weeks. We measured body weight, blood glucose levels, creatinine, blood urea nitrogen (BUN), albumin-creatinine ratio (ACR), kidney injury molecule-1 (KIM-1), oxidative stress, and inflammation. Kidney tissues were also examined for damage and fibrosis markers, particularly transforming growth factor beta-1 (TGF-β1). Diabetic rats exhibited weight loss, hyperglycemia, elevated creatinine, BUN, ACR, KIM-1, increased oxidative stress, and inflammation, along with notable renal damage and higher TGF-β1 expression. Treatment with CVL-SNEDS significantly improved these parameters, reducing blood glucose levels by 51%, creatinine by 39%, BUN by 67%, and ACR by 78%, while also alleviating inflammation and oxidative stress and protecting against kidney damage and fibrosis. These findings suggest that CVL-SNEDS may represent a promising therapeutic approach for slowing DN progression by enhancing glycemic control and mitigating fibrosis, inflammation, and oxidative stress.

**Graphical Abstract:**

Schematic diagram showing the mechanism of SNEDS-loaded CVL as potential treatment strategies for type-1 diabetes mellitus and renal fibrosis. This mechanism includes the reduction of TNF-α & IL-1β expression, antioxidant activity, and activation of the KIM1/TGF-β1/TNF-α signaling cascade.

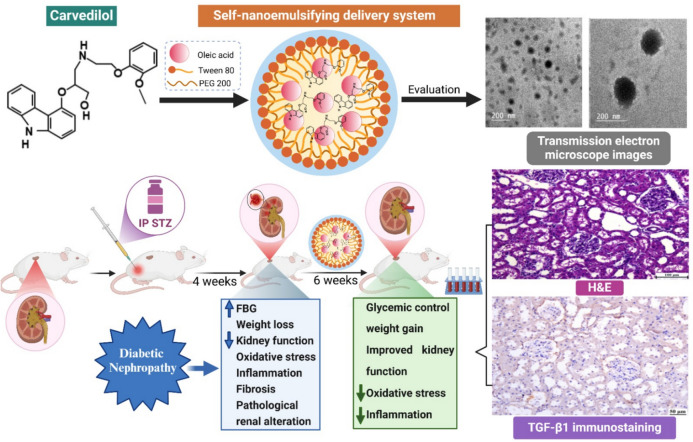

## Introduction

Diabetes mellitus (DM) is a metabolic disorder that forms a major threat to global public health (Kumar et al. 2020). Diabetic complications are considered the leading cause of elevated levels of morbidity and mortality among diabetic patients (Fagherazzi 2023). The International Diabetes Federation (IDF) reported that diabetes was responsible for 6.7 million deaths globally (Magliano and Boyko 2022). Diabetic nephropathy (DN) is one of the macrovascular complications of DM. It is a long-lasting, advancing kidney disease that occurs gradually, typically reaching its highest occurrence rate 10 to 20 years after the onset of diabetes. Diabetes and hypertension are the primary factors contributing to the growing burden of chronic kidney disease (CKD) (Samsu 2021; Kanasaki et al. 2024). DN is more commonly known as diabetic kidney disease (DKD), which accounts for about 30 to 50% of all end-stage renal disease (ESRD) patients worldwide (Wang et al. 2021). DN is estimated to impact around 25 to 35% of those diagnosed with either type 1 DM (T1DM) or type 2 DM (T2DM) (Khoury et al. 2020). Notably, the development of progressive proteinuria is an important hallmark of DN, and properly avoiding and treating proteinuria provides a substantial challenge in DN treatment (Goldstein 2022; Kanasaki et al. 2024). The pathogenesis of DN is multifactorial and complex. It is characterized by significant metabolic perturbations, such as hyperglycemia and dyslipidemia, and hemodynamic perturbations, such as glomerular hypertension. These factors interact with each other to contribute to the development and progression of DN and its progression toward ESRD. Hyperglycemia significantly impacts the development of DKD, leading to an increase in mitochondrial oxidative stress. This increase in oxidative stress is considered the primary mechanism behind the pathophysiology of DKD, although additional pathways have also been suggested, including disrupted intracellular metabolism, inflammation, apoptosis, and tissue fibrosis (Gembillo et al. 2021).

Recently, DN management depends on hyperglycemia control and blood pressure management using angiotensin-converting enzyme (ACE) inhibitors and angiotensin II receptor blockers (ARB). Nevertheless, conventional treatment approaches may not yield satisfactory outcomes in DN management. Although DN poses a significant strain on society and receives worldwide concern, there is a scarcity of medications specifically designed to address the therapy of DN (Xiong and Zhou 2019). Consequently, there has been a growing emphasis on improving innovative approaches for DN management. β-blockers are antihypertensive drugs that are pharmacologically used to manage hypertension. Carvedilol (CVL) 1-(9H-carbazol-4-yloxy) −3-[[2-(2-methoxyphenoxy) ethyl] amino]−2-propanol is an α−1-adrenoreceptor antagonist and one of the third generation of non-selective β-blockers. It has anti-inflammatory, antioxidant, anti-apoptotic, and calcium-antagonist properties. Moreover, it is a vasodilator due to its effect as α−1-adrenoreceptor antagonist; thus, it can decrease peripheral vascular resistance. Furthermore, it has been observed that CVL demonstrates a protective effect in several instances of kidney damage caused by gentamicin (Kumar et al. 2000), cisplatin (Rodrigues et al. 2011), and ischemia–reperfusion (Hayashi et al. 2010). Although CVL is widely used in medical settings, it exhibits low and variable bioavailability due to inadequate absorption and significant hepatic first-pass metabolism (about 25 to 35% bioavailability) (Amarachinta et al. 2021). Therefore, there is a great need to improve CVL solubility and its oral bioavailability to boost its efficacy. This goal can be achieved by formulating CVL in a nanostructured system.

Self-nanoemulsifying drug delivery system (SNEDS) is a leading nanosystem. It is a lipid-based formulation that is composed of a combination of oil, surfactant, and cosurfactant. These concentrated formulations with gentle agitation undergo spontaneous conversion into oil-in-water nano-emulsions when introduced to an aqueous medium (Kadian and Nanda 2023). SNEDS have demonstrated significant advantages beyond enhancing drug solubility and absorption. They also improve drug stability, intestinal permeability, oral bioavailability, and overall pharmacokinetic and therapeutic profiles when compared to conventional drug forms (Morgan 1994; Singh et al. 2011, 2013b; Rathore et al. 2023). Accordingly, the present study was designed to develop a CVL-loaded SNEDS formulation with the aim of achieving controlled drug release and enhanced oral bioavailability, thereby improving its therapeutic efficacy in the management of diabetic nephropathy (DN). The current investigation was structured to compare the disease-modifying potential of conventional CVL to that of the nano-formulated CVL (CVL-SNEDS) in a validated DN model.

## Materials and methods

### Materials

Carvedilol powder was a gift from Riyadh Pharma (Riyadh, Saudi Arabia). Oleic acid, polyethylene glycol (PEG) 200, Tween 80, STZ, thiobarbituric acid (TBARS), hydrochloric acid, ethanol, and trichloroacetic acid were purchased from Sigma-Aldrich (St. Louis, MO, USA). Carboxymethyl cellulose (CMC) and sucrose were purchased from El-Nasr Chemical Industries Co., Cairo, Egypt. Citric acid was purchased from El Gomhouria pharmaceutical company, Cairo, Egypt. Creatinine assay kit (Cat# 501V21.0) and albumin assay kit (Cat# 501V15.0) for the measurement of albumin-creatinine ratio (ACR) were from Roche Diagnostics GmbH, Sandhofer Strasse 116, D-68305 Mannheim. Serum creatinine (Cat# 234 001) and blood urea nitrogen (BUN) (Cat# EIABUN). Kidney injury molecule-1 (KIM-1) (Cat# 80,684—Crystal Chem company, Elk Grove Village, IL, USA). An assay kit for the measurement of levels of reduced glutathione (GSH) (Cat# GR 25 11) and superoxide dismutase (SOD) (Cat# SD 25 21) were purchased from Biodiagnostic for diagnostic and research reagents, Egypt. Tumor necrosis factor-alpha (TNF-α) (Quantikine™ Rat TNF-α ELISA kit, Cat# RTA00—R&D Systems Inc., USA) and interleukin-1 beta (IL1-β) (Invitrogen Rat IL-1β ELISA kit, Cat# BMS630—Thermo Fisher Scientific Inc.). Protein assay kit (Quick Start™ Bradford Protein Assay, Cat# 500–0202—Bio-Rad)*.* The Direct-zol RNA Miniprep Plus Kit (Cat# R2072, Zymo Research Corp., USA). The Invitrogen SuperScript™ IV One-Step RT-PCR kit (Cat# 12,594,100, Thermo Fisher Scientific, Waltham, MA, USA). Anti-transforming growth factor-β1 (TGF-β1) Ab (Cat# FNab08638; Fine Test, China).

### Development of CVL-SNEDS

#### Preparation of CVL-SNEDS

CVL-SNEDS was prepared using the same method as previous studies (Singh et al. 2013a; Ahmed et al. 2018; Aldawsari et al. 2018). In brief, oleic acid was selected as the oil phase, Tween 80 was utilized as a surfactant, and PEG 200 served as a co-surfactant in proportions of 10%, 50%, and 40%, respectively. The three components were combined in an Eppendorf tube and subjected to a 5-min vortexing process with 10 mg of CVL to obtain a 1-g sample of the SNEDS. This was done to completely solubilize the CVL in the formulation components.

#### Evaluation of CVL-SNEDS: visual evaluation, emulsification ability, and thermodynamic stability

The dispersions of CVL-SNEDS were visually examined to assess their tendency for spontaneous emulsification, transparency, and the overall look of the dispersed system. Furthermore, we analyzed the thermodynamic stability and propensity for phase separation of CVL-loaded SNEDS (Ahmed et al. 2018). The SNEDS formulations underwent centrifugation at a speed of 4000 revolutions per minute for 20 min. Additionally, they were exposed to three freezing and thawing cycles, with temperatures ranging from − 20 to + 25 °C (Elimam et al. 2022, 2025). The formulations were subsequently assessed for cracking, creaming, or any signs of phase separation.

#### Robustness to dilution

The characteristics of CVL-SNEDS were assessed after dilution with double-distilled water at ratios of 50, 100, and 1000 times. The diluted nanoemulsions (NEs) were held for 12 h and assessed for indications of drug precipitation or phase separation (Khalid et al. 2017; Elimam et al. 2020).

#### Determination of CVL-SNEDS size and zeta potential

CVL-SNEDS was mixed with 20 mL of distilled water, resulting in dilution. The dispersion was analyzed for hydrodynamic diameter, polydispersity index (PDI), and zeta potential (ZP) using a Malvern Zetasizer Nano ZSP manufactured by Malvern Panalytical Ltd. in the UK. The measurements were conducted at a temperature of 22 °C.

#### Surface morphology of the CVL-SNEDS

The morphological characteristics of CVL-SNEDS were examined using transmission electron microscopy (TEM; 100CX; JEOL, Tokyo, Japan). In TEM analysis, a small amount of diluted CVL-SNEDS formulation was applied onto a microscope grid covered with carbon. After 3 min of drying, it was dyed with a 1% phosphotungstic acid solution and then examined.

### Animals

Adult male Wistar Albino rats weighing 180–200 g were provided by the holding company for biological products and vaccines safe (VACSERA CO.). Animals were grown and kept at the institution’s animal house. Rats were maintained at 25 °C, subjected to a 12-h light/12-h dark cycle, and fed standard pellet chow ad *libitum* throughout the study. All rats had unrestricted access to food and water. This study was performed in accordance with the Ethics Committee of the Faculty of Pharmacy, Helwan University, Egypt.

### Experimental design and induction of diabetes

**Fig. 1:**
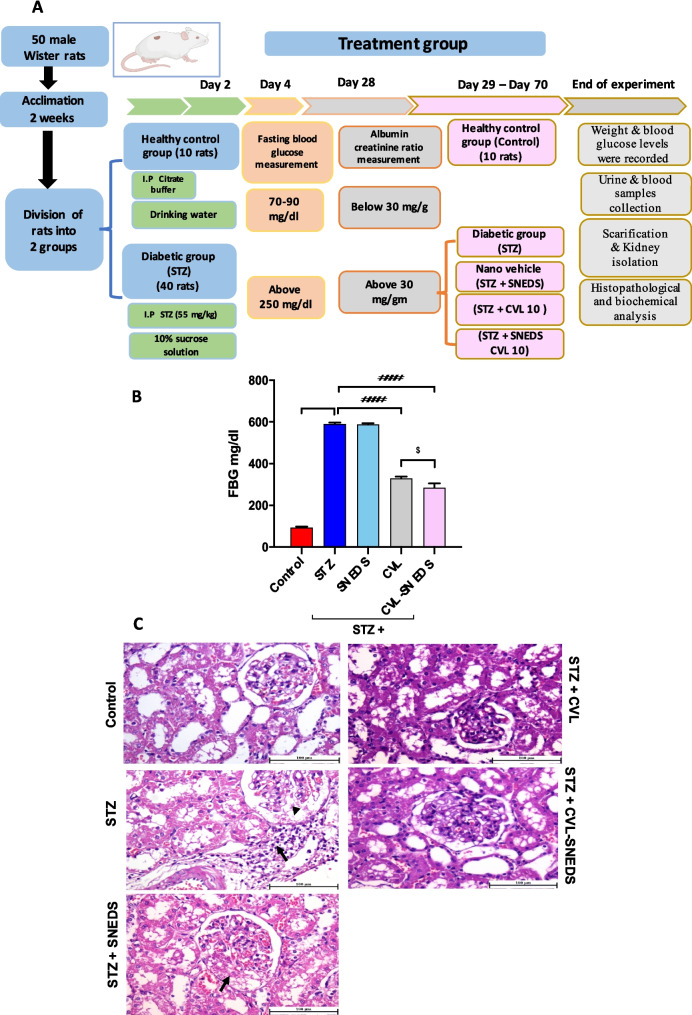
CVL-SNEDS reduced fasting blood glucose. **A** Study design. **B** Bar graph represents fasting blood glucose. There was a significant elevation in FBG in STZ compared to control ****P < 0.0001. The administration of CVL and CVL-SNEDS reduced FBG. CVL and CVL-SNEDS reduced FBG significantly compared to STZ ^####^*P* < 0.0001. CVL-SNEDS significantly improved FBG compared to CVL ^$^*P* < 0.05. **C** Histopathology of the kidney in STZ-induced DN rats. Microscopic examination of the kidney from the control group showed normal renal cortex with healthy glomeruli. The STZ-induced DN group showed periglomerular inflammatory cell infiltration (arrow) with thickening of the glomerular basement membrane (arrowhead) and severe cortical blood vessel congestion and capillary tufts in the renal glomeruli. The STZ-SNEDS group showed leakage in the Bowman’s space in the renal glomeruli (arrow) with a marked increase in glomerular size. Mild congestion in the renal cortex was shown in the STZ + VL group; meanwhile, the STZ + CVL-SNEDS-treated group showed normal renal cortex

Rats were acclimatized for two weeks and then randomly assigned to treatment groups. The treatment group was divided into five subgroups (10 rats per group). The control group received an intraperitoneal injection (IP) of citrate buffer. The diabetic group (STZ) was given a single IP of STZ (55 mg/kg, dissolved in 0.1 M sodium citrate buffer, pH 4.5) to induce DN (Ghasemi and Jeddi 2023) (Tesch and Allen 2007; Furman 2021). The nano-vehicle group (STZ + SNEDS) received both STZ and a nano-vehicle, while another diabetic group (STZ + CVL 10) was treated with 10 mg/kg carvedilol powder (Morsy et al. 2014). A final group (STZ + CVL-SNEDS 10) was treated with a nanoformulation of carvedilol (SNEDS) equivalent to 10 mg/kg. Diabetic rats received STZ after overnight fasting, followed by a 48-h administration of 10% sucrose to prevent hypoglycemia-related mortality. After 72 h, fasting blood glucose (FBG) was measured, and rats with FBG above 250 mg/dL were sorted as diabetic. These rats were untreated for 4 weeks to validate the DN model by measuring the albumin-creatinine ratio (ACR). Upon confirmation of DN (ACR > 30 mg/g), treatment groups received CVL or CVL-SNEDS for 6 weeks. Body weight, blood glucose, urine, and blood samples were monitored, and kidneys were collected for further analysis. Figure 1A illustrates the study design.

### Blood and tissue collections

By the end of the experiment, urine samples were collected using metabolic cages and centrifuged for 10 min at 2000 rpm to eliminate any debris present. Rats were weighed, blood glucose was measured, blood samples were taken in tubes and centrifuged for 10 min at 2000 rpm, and serum samples were taken for further investigations. Finally, the scarification of rats was done by decapitation followed by kidney isolation on ice. Formalin was used to fix kidney tissues, which were then processed and embedded in paraffin using standard methods for histopathological and immunohistochemical analysis. Other kidney tissues were kept frozen ^◦^at − 80 °C for further investigations.

### Histopathological examination

The fresh kidney samples were collected and kept in 10% neutral buffered formalin, then dehydrated using ethanol in different concentrations. Afterward, the samples were treated with xylene to remove impurities and placed in paraffin wax for preservation. They were then cut into sections of 5 µm thickness and stained with hematoxylin & eosin (H & E) for subsequent histopathological examination (Bancroft 2013). Tissue slides were analyzed with an Olympus BX43 light microscope and captured using an Olympus DP27 camera connected to Cellsens Dimensions software (Olympus). Changes in the experimental histopathologic glomerular damage for kidney tissue were graded as follows: (0) showing no damage, (1), (2), and (3) indicating mild, moderate, and severe glomerular damage, respectively (Medeiros et al. 2010). Data was statistically analyzed by non-parametric analysis (Kruskal–Wallis test) to test the significance difference between groups compared to the control group (Aydin et al. 2003).

### Biochemical analysis

#### Colorimetric assays

Blood urea nitrogen (BUN) and serum creatinine were assessed spectrophotometrically as indicators of renal function. To evaluate proteinuria, we calculated the urine albumin/creatinine ratio using the following equation: [ACR = albumin (mg/dl)/creatinine (g/dl)] (Febrianto et al. 2020). Oxidative stress and antioxidant markers, including GSH and SOD, were assessed in the kidney homogenates utilizing the previously mentioned kits, and all procedures were done in compliance with the manufacturer’s instructions. Malondialdehyde (MDA) was also measured in kidney homogenate using the thiobarbituric acid (TBA) assay method; 100 µl of the tissue homogenate was mixed with 400 µl of distilled water, 30 µl of butylated hydroxytoluene (PHT), and 1 ml of TBA reagents. Subsequently, it was maintained in a water bath at 90 °C for 15 min and then cooled and centrifuged at 2200 rpm for 10 min at 4 °C. The supernatant was collected to assess the absorbance at 532 nm. The MDA concentration (mmol/mg protein) was calculated according to the following equation (Kheradmand et al. 2009).

$$\text{MDA }(\text{m}.\text{mol}/\text{mg protein})=\frac{\text{Absorbance}}{\left(1.56\times \text{mg protein in the sample}\right)}\times {10}^{5}$$

#### Determination of TNF-α, IL1-β, and KIM-1 by enzyme-linked immunosorbent assay (ELISA)

The protein content in the tissue homogenates was quantified using the Bradford (Bradford 1976) method to calculate oxidative stress and inflammatory biomarkers per milligram of protein. The renal levels of inflammatory cytokines TNF-α and IL1-β were quantified using ELISA following the manufacturer’s protocol. Additionally, KIM-1 was assessed as a serum indicator of renal tissue injury.

#### Total RNA extraction and real-time qPCR for analysis of inducible nitric oxide synthase 2 (iNOS2) gene expression

Following the manufacturer’s protocol, the Direct-zol RNA Miniprep Plus Kit was used for total RNA extraction from isolated kidney tissues. The quantity and purity of extracted RNA were evaluated using a NanoDrop™ One Microvolume UV–Vis spectrophotometer (Thermo Fisher Scientific). The extracted RNA was reverse-transcribed using the Invitrogen SuperScript ™ IV One-Step RT-PCR kit, and then PCR was performed. Using a 96-well plate StepOne instrument, a thermal profile was as follows: reverse transcription takes place for 10 min at 45 °C, RT inactivation takes place for 2 min at 98 °C, initial denaturation by 40 cycles of 10 s at 98 °C, and the amplification phase takes place for 10 s at 55 °C and 30 s at 72 °C. Data were expressed in the cycle threshold (Ct) for both the housekeeping and target genes following the RT-PCR run (Gauvin et al. 2023). Duplicate analysis was performed. Normalization for variability in the inducible nitric oxide synthase 2 gene (iNOS2; QT00178325) expression was analyzed using the mean critical threshold (CT) values of the glyceraldehyde-3-phosphate dehydrogenase (GAPDH; QT00199633) housekeeping gene, employing the ΔΔCt method. The target gene’s relative quantification (RQ) was calculated using the 2^−ΔΔCT^ technique (Elimam et al. 2024). All primers utilized in the experiment were pre-prepared QuantiTect® Primer Assay kits acquired from Qiagen.

#### Immunohistochemical analysis

Briefly, slides were deparaffinized and rehydrated for immunohistochemical detection of the fibrotic marker TGF-β1 expression. Slides were then transferred to the antigen retrieval solution, followed by overnight antibody incubation with rabbit polyclonal anti-TGF-β1 Ab at a dilution of 1:200 at 4 °C. Incubation with the optimum secondary antibody and chromogen was carried out in accordance with the manufacturer’s guidelines. Finally, signal detection and quantification of the images were done using the ImageJ software (USA).

### Statistical analysis

GraphPad Prism statistical software, version 8, was used to analyze data. All data were presented as mean ± SEM. The ordinary one-way ANOVA for multiple comparisons was followed by Welch’s test to compare statistical differences and determine the significance level between groups. Statistical significance was defined as a *P* value < 0.05.

## Results

### Evaluation of the CVL-SNEDS formulation

CVL-loaded SNEDS demonstrated no creaming, cracking, or phase separation. Additionally, there was negligible variation in globule diameters before and after three cycles of freezing and thawing at temperatures of − 20 °C and + 25 °C. These results indicate a high level of thermodynamic stability for the formulations.

**Fig. 2:**
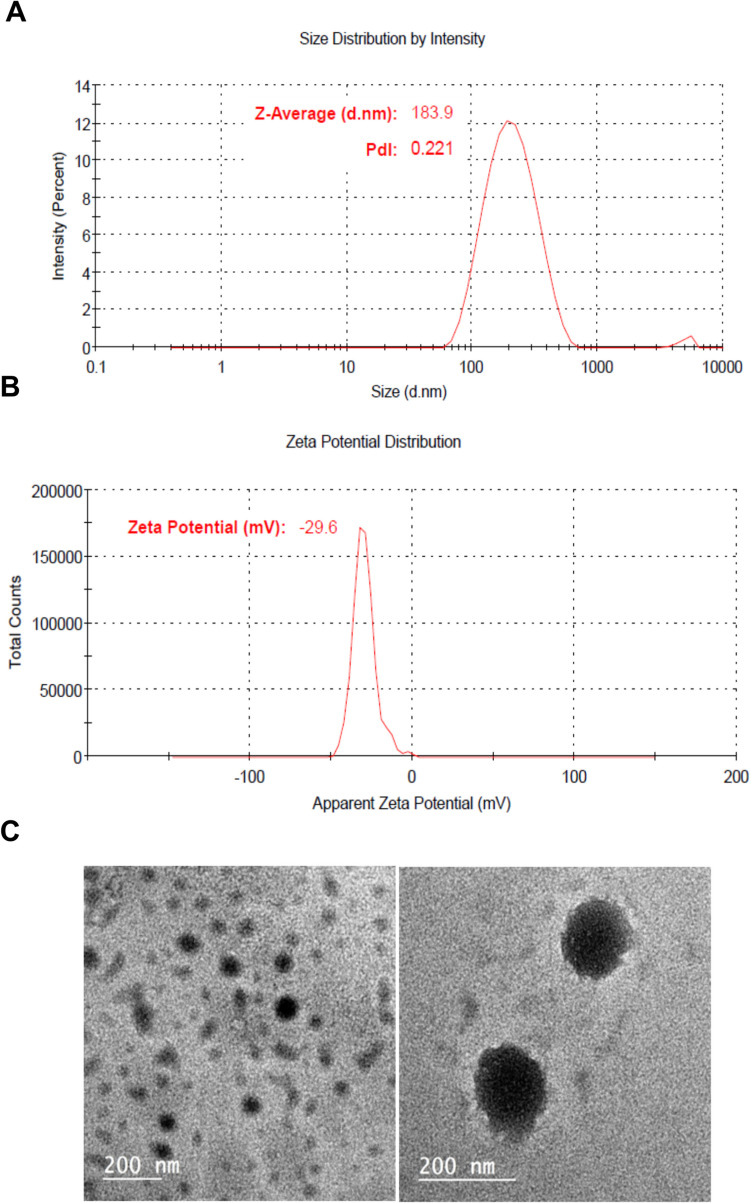
Graphical representation of particle size (**A**), zeta potential (**B**), and transmission electron microscope images (**C**) of the CVLSNEDS formula

The NEs created by diluting CVL-SNEDS with distilled water remained very stable at all dilution levels, with no phase separation or drug precipitation observed even after 24 h of storage. The droplet size of CVL-SNEDS exhibited a unimodal distribution, with an average size of 183.9 nm and a low polydispersity index (PDI) value of 0.221 (Fig. 2A). This limited range of sizes is crucial for determining drug release in laboratory settings, as well as for absorption into living organisms and uptake into biological systems (Limbach et al. 2005; Shi et al. 2008). The ζ-potential was − 29.6 mV (Fig. 2B), indicating that the solution remained stable upon dilution.

Furthermore, transmission electron microscopy (TEM) images (Fig. 2C) revealed that the colloidal dispersion in an aqueous solution exhibits a spherical particle shape, suggesting that CVL-SNEDS generates uniform nanoscale structures. Visual evidence confirms the consistency of the size distribution.

### CVL and CVL-SNEDS improved weight loss and reduced blood glucose levels in STZ-diabetic rats

Rats with matched body weights (BW) were selected at the beginning of the experimental study, and no significant differences were observed between the groups. At the end of the experiment, both the diabetic group (STZ) and the diabetic group receiving the nano-vehicle (STZ + SNEDS) exhibited a significant decrease in BW (Table 1) and an increase in fasting blood glucose (FBG) compared to the control group (Fig. 1B). However, treatment with 10 mg/kg of CVL powder (STZ + CVL) and the nanoformulation of CVL at an equivalent dose of 10 mg/kg BW (STZ + CVL-SNEDS) resulted in a significant gain in BW and a decrease in FBG compared to the diabetic group. Furthermore, CVL-SNEDS demonstrated a significant reduction in blood glucose levels compared to the CVL group, as shown in Fig. 1B.

**Table 1:** Body weight in treatment groups

Experimental groups	Control	STZ	STZ + SNEDS	STZ + CVL	STZ + CVL-SNEDS
Body weight day 1 (gm)	191.50 ± 3.10	190.33 ± 2.45	191.50 ± 2.75	195.83 ± 1.99	193 ± 2.35
Body weight at the end of the experiment (gm)	220.00 ± 3.204	164.50 ± 2.742^****^	165.67 ± 2.704^****^	190.83 ± 1.990 ^####^	193.33 ± 2.362 ^####^

Data are represented as mean ± SEM. ****, ####: *P* < 0.0001 compared to the control group and STZ group respectively (ANOVA). Six rats per group.

### CVL and CVL-SNEDS effect on renal histopathology

Microscopic examination of the kidneys from the control group revealed a normal renal cortex and medulla, with intact renal glomeruli and no visible histological alterations. In contrast, the STZ and STZ + SNEDS groups exhibited increased glomerular size in the affected renal cortex, accompanied by thickening of the glomerular basement membrane and infiltration of inflammatory cells around the glomeruli. Severe congestion of peritubular blood vessels was also observed in the affected renal tissue, along with congested capillary tufts in the renal corpuscles. Thickening of the mesangial matrix was noted in several affected glomeruli. In addition, a semiquantitative analysis such as histopathological scoring for glomerular damage revealed significant differences in the STZ and STZ + SNEDS groups compared to the control group. The CVL-treated group was significantly different from the control group. Moderate improvement was observed in the STZ + CVL-treated group, where the renal tissue showed variable interstitial nephritis and congested blood vessels, while cystically dilated renal tubules were detected less frequently. The most significant improvement was noted in the STZ + CVL-SNEDS group as the histopathological scoring for glomerular damage of this group exhibited no significance compared to the control group (Table 2 and Fig. 1C).

**Table 2:** Histopathological Scoring for glomerular damage

Experimental groups	Control	STZ	STZ + SNEDS	STZ + CVL	STZ + CVL-SNEDS
glomerular damage Score	0.00 ± 0.00	3.00 ± 0.00^****^	3.00 ± 0.00^****^	2.17 ± 0.16^*^	1.00 ± 0.00

Data are represented as mean ± SEM. ****P < 0.0001 and *P < 0.05 compared to the control group (Kruskal–Wallis test). Six rats per group.

### CVL and CVL-SNEDS improved kidney function in STZ-diabetic rats

**Fig. 3:**
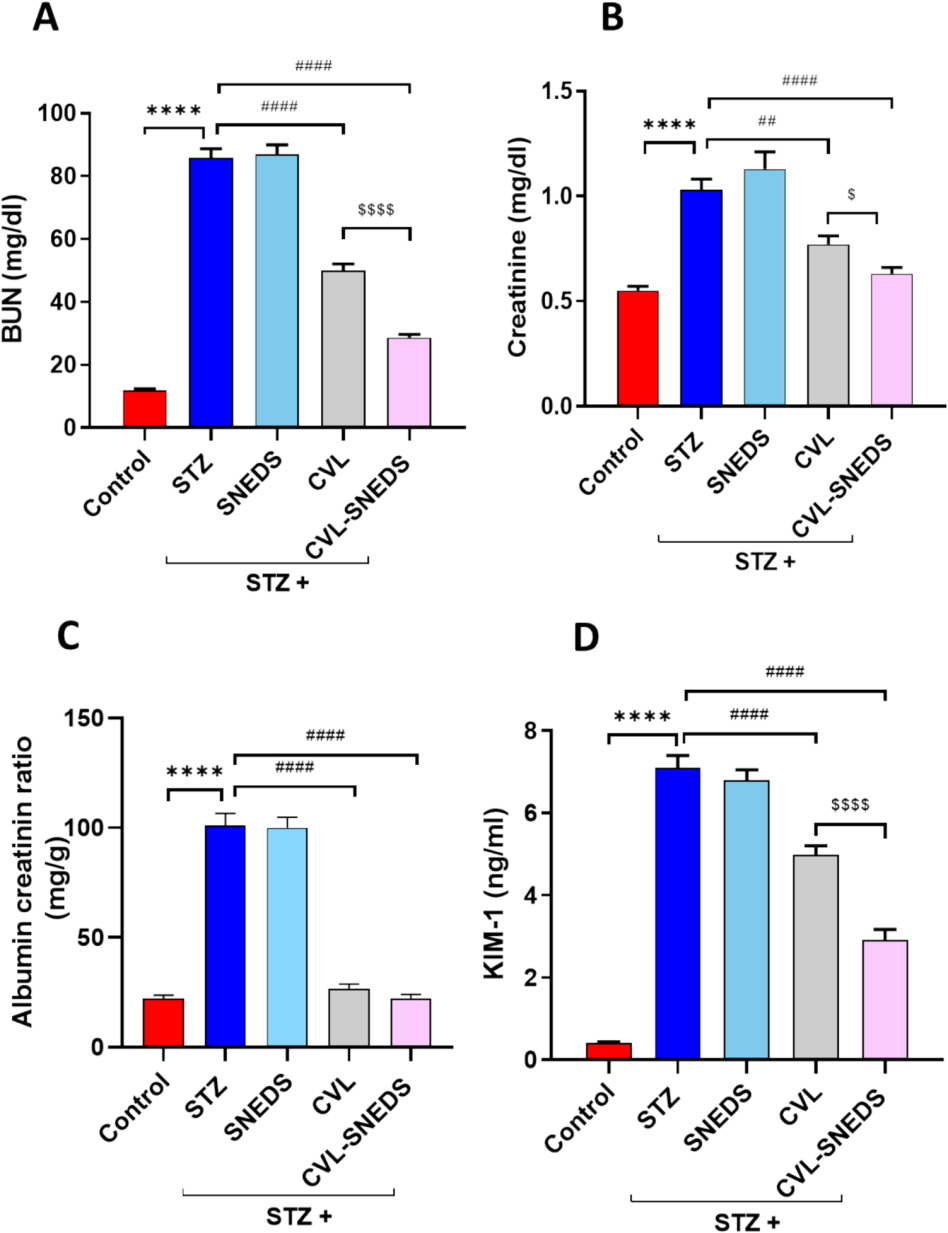
Effect of CVL and CVL-SNEDS on kidney function indicators: **A** STZ and STZ + SNEDS increased BUN level compared to control group ****P < 0.0001. CVL and CVL-SNEDS decreased BUN level compared to STZ group ^####^*P* < 0.0001. A significant decrease in BUN level was observed in CVL-SNEDS compared to CVL ^$$$$^*P* < 0.0001. **B** STZ and STZ + SNEDS increased serum creatinine level compared to control group ****P < 0.0001. CVL and CVL-SNEDS decreased creatinine level compared to STZ ^##^*P* < 0.01, ^####^*P* < 0.0001. A significant decrease in creatinine level was observed in CVL-SNEDS compared to CVL ^$^*P* < 0.05. **C** STZ and STZ + SNEDS increased ACR level compared to control group ****P < 0.0001. CVL and CVL-SNEDS decreased ACR ratio compared to STZ ^####^*P* < 0.0001. **D** STZ and STZ + SNEDS increased KIM-1 level compared to control group ****P < 0.0001. CVL and CVL-SNEDS decreased KIM-1 level compared to control ^####^*P* < 0.0001. A significant decrease in CVL-SNEDS KIM-1 level compared to CVL ^$$$$^*P* < 0.0001. There were six rats per group

BUN, creatinine, ACR, and KIM-1 levels were measured to assess kidney function. There was a significant increase in BUN and creatinine levels in the diabetic groups compared to the control group. However, BUN and creatinine levels declined in the CVL and CVL-SNEDS treated groups compared to the diabetic groups. Since ACR is the primary method for assessing kidney function in DN patients (Sagoo and Gnudi 2020), urine samples were utilized to measure ACR. Our results revealed a significant increase in ACR in the diabetic group (STZ) compared to the control group. The treated groups with CVL and CVL-SNEDS showed significant decreases in ACR compared to the diabetic groups (73% and 78%, respectively), as shown in Fig. 3.

Additionally, KIM-1 levels in the diabetic groups were significantly elevated compared to the control group. However, there was a notable decline in KIM-1 levels in the CVL and CVL-SNEDS-treated groups compared to the STZ group (30% and 59%, respectively). Interestingly, CVL-SNEDS significantly reduced KIM-1 levels by 41% compared to the CVL-treated group.

### CVL and CVL-SNEDS effect on oxidative stress in STZ-diabetic rats

**Fig. 4:**
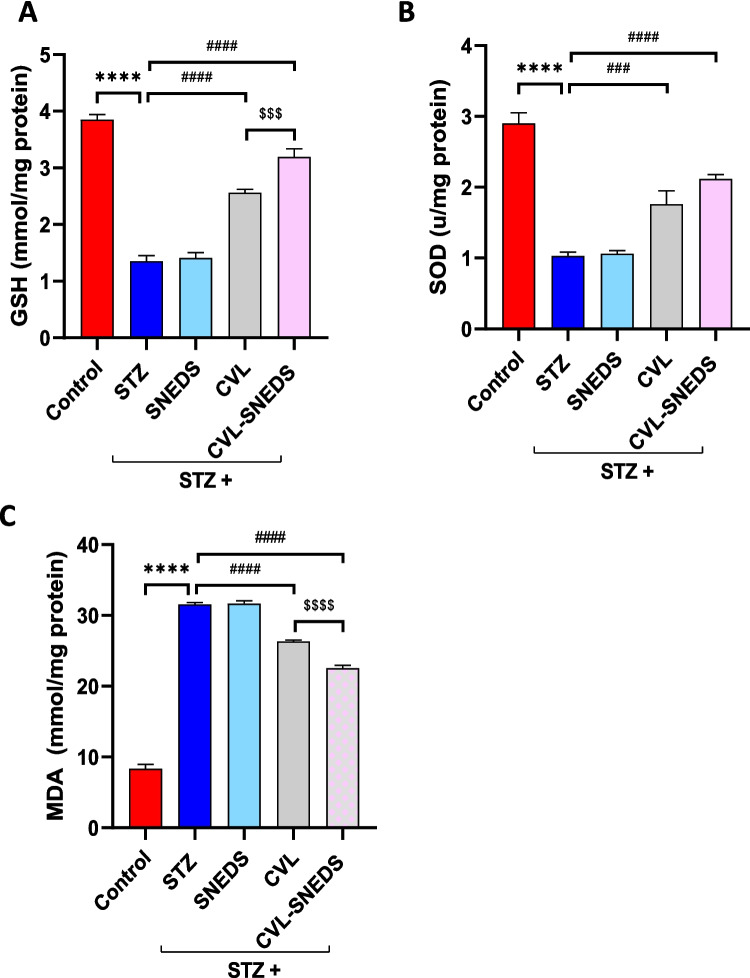
Effect of treatment with CVL and CVL-SNEDS on oxidative stress: **A** STZ and STZ + SNEDS decreased GSH level compared to control group ****P < 0.0001. Treatment with CVL and CVL-SNEDS increased GSH level compared to STZ ^####^*P* < 0.0001. A significant increase in GSH level was observed in CVL-SNEDS compared to CVL ^$$$^*P* < 0.001. **B** STZ and STZ + SNEDS decreased SOD level compared to control group ****P < 0.0001. Treatment with CVL and CVL-SNEDS increased SOD level compared to STZ ^###^*P* < 0.001, ^####^*P* < 0.0001 **C.** STZ and STZ + SNEDS increased MDA level compared to control group ****P < 0.0001. Treatment with CVL and CVL-SNEDS decreased MDA level compared to STZ ^####^*P* < 0.0001. A significant decrease in MDA level was observed in CVL-SNEDS compared to CVL ^$$$$^*P* < 0.0001. There were six rats per group

As the progression of DN is mainly driven by oxidative stress (Khoury et al. 2020; Jin et al. 2023b), we assessed the levels of GSH and SOD activity in renal tissue. Both GSH and SOD levels in the diabetic groups were significantly lower than those in the control group. In the treated groups with CVL or CVL-SNEDS, SOD and GSH levels were significantly elevated by approximately 2- to 2.3-fold compared to the diabetic groups. Additionally, GSH levels in the STZ + CVL-SNEDS-treated group increased significantly by 24% compared to the STZ + CVL-treated group, as shown in Fig. 4.

Furthermore, MDA levels were measured in kidney homogenate. MDA concentrations in the diabetic groups were significantly elevated compared to those in the control group. Both treatment groups exhibited a significant decrease in MDA levels compared to the diabetic groups. Notably, the STZ + CVL-SNEDS group showed a 14% reduction in MDA compared to the STZ + CVL-treated group (Fig. 4C).

### CVL and CVL-SNEDS effect on renal inflammatory markers

**Fig. 5:**
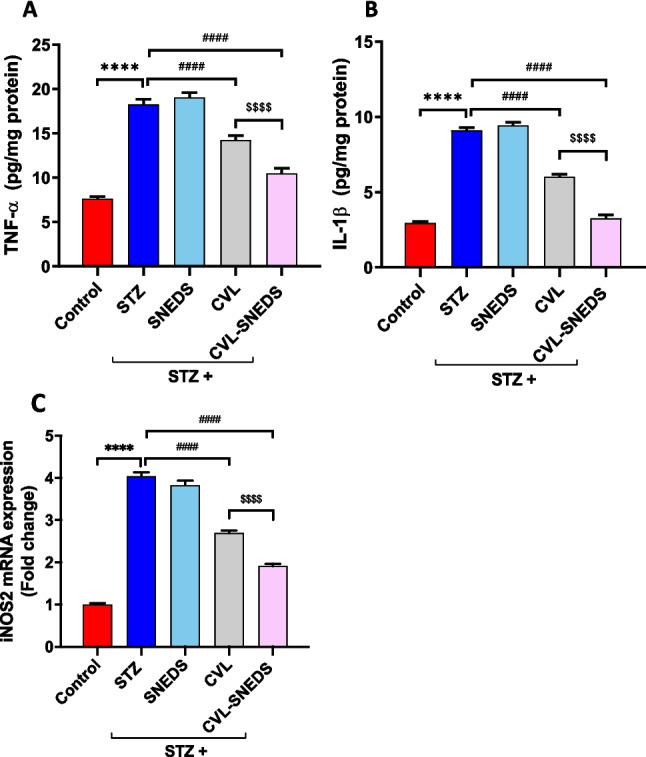
Effect of treatment with CVL and CVL-SNEDS on inflammation. **A** STZ and STZ + SNEDS increased TNF-α level compared to control group ****P < 0.0001. Treatment with CVL and CVL-SNEDS decreased TNF-α level compared to STZ ^####^*P* < 0.0001. A significant decrease in TNF-α level was observed in CVL-SNEDS compared to CVL ^$$$$^*P* < 0.0001. **B** STZ and STZ + SNEDS increased IL-1β level compared to control group ****P < 0.0001. Treatment with CVL and CVL-SNEDS decreased IL-1β level compared to STZ ^####^*P* < 0.0001. A significant decrease in IL-1β level was observed in CVL-SNEDS compared to CVL ^$$$$^*P* < 0.0001. **C** STZ and STZ + SNEDS increased iNOS2 expression compared to control group ****P < 0.0001 Treatment with CVL and CVL-SNEDS decreased iNOS2 expression compared to STZ ^####^*P* < 0.0001. A significant decrease in iNOS2 expression was observed in CVL-SNEDS compared to CVL ^$$$$^*P* < 0.0001. There were 6 rats per group

Inflammatory cytokines (TNF-α and IL-1β) and the RT-PCR mRNA expression level of inducible nitric oxide synthase (iNOS2) were measured. The results indicated a significant increase in inflammatory markers in the diabetic groups compared to the control group. In contrast, there was a notable decrease in these markers in the CVL and CVL-SNEDS-treated groups compared to the diabetic groups. Interestingly, the CVL-SNEDS group exhibited significant reductions in TNF-α, IL-1β levels, and iNOS2 gene expression (26%, 45%, and 29%, respectively) compared to the CVL group (Fig. 5).

### CVL and CVL-SNEDS effect on renal fibrotic marker TGF-β1

**Fig. 6:**
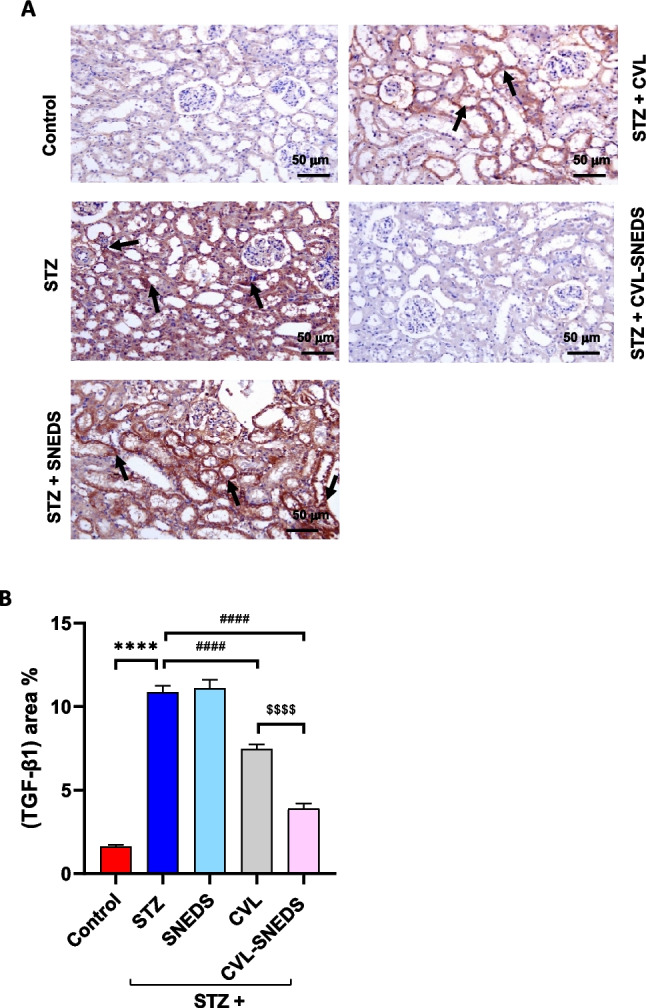
Effect of treatment with CVL and CVL-SNEDS on renal TGF-β1. **A** Photomicrographs of the kidney immunostained with TGF-β1. The control group showed weak expression of TGF-β in the examined renal cortex and medulla; STZ and STZ + SNEDS groups showed strong positive expression as brown staining (arrow). The STZ + CVL group displayed increased expression as brown staining (arrow); adversely, limited levels of TGF-β1 expression were noticed in the STZ + CVL-SNEDS group. Bar = 50 μm. **B** The bar chart represents the TGF-β1 immunopositivity expressed as area %. Mean values with different superscripts are significantly different ^****^P < 0.0001 STZ and STZ-SNEDS treated groups versus control. ^####^*P* < 0.0001 CVL and CVL-SNEDS versus STZ. ^$$$$^*P* < 0.0001 CVL-SNEDS versus CVL group. (ANOVA)

Examination of the renal tissue from the control group revealed weak expression of TGF-β in both the renal cortex and medulla. In contrast, strong positive expression was detected in the STZ and STZ + SNEDS groups. The STZ + CVL-treated group also showed increased expression; however, the STZ + CVL-SNEDS group exhibited limited levels of TGF-β expression (Fig. 6A).

Quantitative analysis of the percentage of TGF-β positive area was conducted across the different experimental groups. Notably, the CVL-SNEDS group demonstrated a significant decrease in TGF-β levels, with a reduction of 48% compared to the CVL group (Fig. 6B).

## Discussion

The primary cause of ESRD is DN, which is fatally impeded by DM. Generally, DN affects around 15–25% of individuals with T1DM and 30–40% of those with T2DM (Magliano and Boyko 2022). This study aimed to formulate CVL-SNEDS and to detect the effect of the regular form of CVL against the CVL-SNEDS on STZ-induced DN in rats. In this study, we addressed the effect of CVL-loaded SNEDS as a potential therapeutic drug in the management of DN. We demonstrate that CVL-SNEDS generates uniform nanoscale structures and exhibits a limited range of sizes, and CVL-SNEDS showed a significant gain in BW and decrease in FBG compared to the diabetic group. In comparison to STZ-treated animals, CVL-SNEDS significantly reduced kidney function. It also decreased BUN, creatinine, ACR, and KIM-1 levels compared to the CVL-treated group. Moreover, CVL-SNEDS elevated GSH and SOD levels and decreased MDA levels compared to the CVL-treated group. Furthermore, the CVL-SNEDS formula was found to reduce TNF-α, IL-1β, and iNOS-2 levels compared to the CVL-treated group. The TGF-β levels in the CVL-SNEDS-treated rats were less than the TGF-β levels in animals treated with CV by 48%.

The DM model was established through STZ administration, which destroys pancreatic β-cells, leading to DM and its associated complications, including DN. Common symptoms of T1DM include polyuria, polydipsia, polyphagia, unintentional weight loss, visual disturbances, and fatigue. STZ-induced DM often results in a notable decrease in body weight, primarily due to increased muscle atrophy and tissue protein loss (Wang-Fischer and Garyantes 2018; Alaofi 2020). Diabetic rats that received only SNEDS exhibited significant unintentional weight loss compared to the control group. In contrast, the treatment groups (CVL and CVL-SNEDS) showed significant weight gain compared to the STZ group. These results were consistent with another study (Zheng et al. 2019), which also reported a significant decrease in weight in a diabetic group induced by STZ following a high-fat diet for 4 weeks. Additionally, significant weight gain was observed in groups treated with CVL at a dose of 10 mg/kg/day for 20 weeks in a diabetic cardiomyopathy model. The outcomes of our study regarding weight were consistent with other studies (ALTamimi et al. 2021, Guo et al. 2021, Zhu et al. 2021, Hu et al. 2022, Alshehri 2023) that documented weight loss in diabetic rats. This weight loss is indicative of T1DM symptoms and supports the validation of the diabetic model.

Moreover, CVL was found to impact significantly the glycemic status in our study. CVL and CVL-SNEDS reduced FBG significantly in treated rats compared to diabetic ones, and CVL-SNEDS showed more reduction in FBG compared to the CVL group. This anti-hyperglycemic effect of CVL–SNEDS also supports its effect on improving weight loss. These outcomes are also in harmony with a prior study (Amirshahrokhi and Zohouri 2021) showing that CVL effectively decreased pancreatic β-cell damage and the occurrence of STZ-induced T1D in a dose-dependent manner and effectively inhibited the infiltration of lymphocytes and insulitis in the pancreatic tissue of mice. The effect of CVL on glycemic control was also supported by another research, which indicates that the action of CVL on various K + channels may have a good impact on glucose homeostasis, hence contributing to its therapeutic efficacy in treating hypertensive individuals with T2DM (Li 2022).

DN may remain asymptomatic for the whole lifespan of a small subset of people who have modest diabetes-induced renal impairment. Several biomarkers are available for early detection of DN, including BUN and creatinine, which are tested for initial diagnosis of kidney disease (Chen et al. 2020). In the current study, STZ-induced DN increased BUN and creatinine levels, but treatment with CVL and CVL-SNEDS significantly ameliorated kidney function. From a clinical perspective, DN is often identified by a gradual rise in the amount of albumin excreted in the urine. As urinary ACR is the most valid test for albuminuria estimation and kidney function evaluation for patients and animals [40], we assessed ACR as a potential early indicator of DN. The results showed that the diabetic group’s ACR is significantly elevated compared to that of the control group. However, a significant decline in ACR was observed in rats treated with CVL and CVL-SNEDS. In addition, KIM-1 is a protein that is overexpressed in the proximal tubule of the kidney after ischemia or toxic renal damage. KIM-1 is recognized as a highly sensitive and specific biomarker for DN and renal damage (Han et al. 2002; Khonsha et al. 2024). The current study revealed elevation of KIM-1 in diabetic groups; however; KIM-1 level was significantly declined with CVL and CVL-SNEDS treatment. These findings are in accordance with prior research (Alzahrani et al. 2020; Tang et al. 2020; Aboismaiel et al. 2024) that have demonstrated a substantial increase in kidney function parameters, including urine protein, creatinine, ACR, blood urea nitrogen, and the kidney index, in the STZ group compared to the control group. In addition, other clinical studies (Memmos et al. 2023; Ahmed et al. 2024) were in harmony with our study, as they also revealed that serum levels of KIM-1 were elevated in the case of DN. Moreover, there was a positive correlation between the duration of diabetes and KIM-1 serum level. Therefore, KIM-1 should not be used only as a DN biomarker for early detection but also as a marker for DN prognosis. Elevation in kidney function in the STZ group was also supported by our histopathological results, which revealed several pathological alterations in different kidney compartments in the STZ group. However, the decline in kidney function biomarkers after receiving CVL & CVL-SNEDS implies their positive effects on renal function. Moreover, the CVL-SNEDS formulation exhibits superior renoprotective effects relative to the standard form of CVL.

Reactive oxygen species (ROS) influence the kidneys by changing hemodynamic and metabolic parameters such as increasing angiotensin II levels, triggering lipid peroxidation, prevailing antioxidants, and initiating cellular inflammation and death. GSH and SOD are antioxidant enzymes suppressed by aldose reductase in cases of hyperglycemia (Sagoo and Gnudi 2018; Ricciardi and Gnudi 2021; Sun et al. 2023). The current study is consistent with these findings by demonstrating the involvement of ROS in the pathophysiology of DN, as demonstrated by significantly diminished GSH and SOD and elevated MDA levels in the kidneys of diabetic rats compared to the control group. Previous research suggests potent antioxidant protection of CVL against myocardial infarction [51], silicosis [52], pancreatic tissue damage [41], and nephrotoxicity (Sahu et al. 2014). Herein, we have confirmed the CVL antioxidant effect on STZ-induced DN, as rats treated with CVL and CVL-SNEDS revealed a significant increase in GSH and SOD and a significant decrease in MDA compared to the diabetic group. Furthermore, the CVL-SNEDS-treated group exhibited significant alterations in both GSH and MDA levels compared to the CVL-treated group, underscoring the improved antioxidant efficacy of the NE formulation.

In addition to oxidative stress, inflammation plays a crucial role in the pathophysiology of DN. Multiple studies demonstrate that the activation of inflammatory signaling is essential to the progression of DN (Jin et al. 2023a). In comparison to non-diabetic individuals, diabetic patients exhibit elevated levels of inflammatory markers, including TNF-α and IL-1β. Inducible NOS is not continuously present in cells, but it is produced when the cell is triggered or stimulated, generally by pro-inflammatory cytokines. The data in the present study were in harmony with our research results, which indicated elevated levels of TNF-α, IL-1β, and iNOS2 in STZ-induced DN rats. We found that CVL and CVL-SNEDS therapy markedly decreased the levels of pro-inflammatory cytokines and iNOS2. In addition, the CVL-SNEDS group was significantly different from CVL in lowering TNF-α, IL-1β levels, and iNOS2 gene expression. Thus, CVL-SNEDS exhibits superior anti-inflammatory activity. Interstitial fibrosis remains the most common final mechanism in most CKD cases. DN is a primary contributor to CKD and constitutes the majority of ESKD. The multifunctional cytokine TGF-β1 is an important part of ECM accumulation, glomerulosclerosis, and kidney fibrosis. Previous studies demonstrated that TGF-β1 production is elevated in kidney tissue and urine throughout the progression of DN (Rauchman and Griggs 2019; Zhang et al. 2021). These findings agreed with our experimental outcomes that revealed a significant elevation in TGF-β1 expression in the STZ group. On the other hand, the CVL and CVL-SNEDS treatment groups significantly showed decreased expression of TGF-β1 compared to the STZ group. Furthermore, CVL-SNEDS produced a substantial decrease compared to the CVL group. Therefore, CVL-SNEDS is more effective as antifibrotic than CVL. The TGF-β1 level was found to be influenced by hyperglycemia management or the administration of drugs that inhibit the renin-angiotensin II–aldosterone system (Rauchman and Griggs 2019; Zhao et al. 2020; Zhang et al. 2021). These previous outcomes provide a guide for explaining one of the mechanisms by which CVL affects TGF-β1 as our research confirmed that CVL improved hyperglycemia. Thus, CVL presumably affects TGF-β1 levels through its direct effect on glycemic control. Another alternative mechanism could be indirectly through KIM-1 expression. KIM-1 is expressed in proximal tubules of humans with diabetic kidney disease. A previous report showed that KIM-1 mediates proximal tubular uptake of palmitic acid-bound albumin, leading to enhanced tubular injury with DNA damage, interstitial inflammation, and fibrosis (Mori et al. 2021). Together, our findings support that CVL and CVL-SNEDS ameliorate renal fibrosis and inflammation that improve hyperglycemia via KIM-1 activity. Our results also support that CVL-SNEDS is much more effective than CVL concerning its antifibrotic effect.

## Conclusion

In summary, our study findings indicated that CVL-SNEDS might be a potential treatment for delaying DN development. Moreover, CVL-SNEDS was discovered to be more powerful than CVL, as it improves hyperglycemia, restricts the synthesis of mesangial cell proteins in the ECM induced by elevated glucose concentrations, and inhibits glomerular hypertrophy, reduces BUN, creatinine, ACR, KIM-1, inflammation, oxidative stress, and the levels of TGF-β1 in the glomeruli of treated groups. Consequently, CVL-SNEDS is an excellent renoprotective agent and a prospective therapeutic strategy for DN.

## Data Availability

All source data for this work (or generated in this study) are available upon reasonable request.

## Abbreviations

DM: Diabetes mellitus
DN: Diabetic nephropathy
CKD: Chronic kidney disease
ESRD: End-stage renal disease (ESRD)
DKD: Diabetic kidney disease
T1DM: Type 1 DM
T2DM: Type 2 DM
ACE: Angiotensin-converting enzyme
ARB: Angiotensin II receptor blockers
CVL: Carvedilol (CVL)
SNEDS: Self-nano-emulsifying delivery system
PEG 200: Polyethylene glycol 200
STZ: Streptozotocin
TBA: Thiobarbituric acid
BHT: Butylated hydroxytoluene
CM: Carboxy methyl cellulose
ACR: Albumin-creatinine ratio
GSH: Glutathione
SOD: Superoxide dismutase
IL1-β: Interleukin-1 beta
TGF-β1: Transforming growth factor beta-1
Nes: Nanoemulsions
PDI: Polydispersity index
TEM: Transmission electron microscopy
BW: Body weight
ZP: Zeta potential
FBG: Fasting blood glucose
WHO: World health Organization
ROS: Reactive oxygen species
ESM: Extracellular matrix
